# The genome sequence of the Brown House-moth, *Hofmannophila pseudospretella* (Stainton, 1849)

**DOI:** 10.12688/wellcomeopenres.19394.1

**Published:** 2023-05-31

**Authors:** Douglas Boyes, Peter W.H. Holland, University of Oxford and Wytham Woods Genome Acquisition Lab, Darwin Tree of Life Barcoding collective, Wellcome Sanger Institute Tree of Life programme, Wellcome Sanger Institute Scientific Operations: DNA Pipelines collective, Tree of Life Core Informatics collective, Darwin Tree of Life Consortium

**Affiliations:** 1 UK Centre for Ecology & Hydrology, Wallingford, England, UK; 2 University of Oxford, Oxford, England, UK

**Keywords:** Hofmannophila pseudospretella, Brown House-moth, genome sequence, chromosomal, Lepidoptera

## Abstract

We present a genome assembly from an individual male *Hofmannophila pseudospretella* (the Brown House-moth; Arthropoda; Insecta; Lepidoptera; Oecophoridae). The genome sequence is 406.2 megabases in span. Most of the assembly is scaffolded into 28 chromosomal pseudomolecules, including the Z sex chromosome. The mitochondrial genome has also been assembled and is 15.5 kilobases in length.

## Species taxonomy

Eukaryota; Metazoa; Ecdysozoa; Arthropoda; Hexapoda; Insecta; Pterygota; Neoptera; Endopterygota; Lepidoptera; Glossata; Ditrysia; Gelechioidea; Oecophoridae; Oecophorinae; *Hofmannophila*; *Hofmannophila pseudospretella* (Stainton, 1849) (NCBI:txid572861).

## Background

The ability to digest the cysteine-rich fibrous protein keratin found in hair, skin and feathers is found in few Lepidoptera. The best-known examples are some members of the Tineidae or ‘Clothes moths’, whose larvae are pests of woollen fabrics. A second example is the ‘Brown House-moth’ *Hofmannophila pseudospretella*, a species belonging to a different taxonomic family, the Oecophoridae. *H. pseudospretella* is a cosmopolitan species originally found in Asia, but which has now spread around the world reaching the United States, Europe and Australia. First recorded in Europe in the 1840s, *H. pseudospretella* can be found in woodland and grassland habitats, but it is also a synanthropic species living inside homes and warehouses. It has been recorded across Britain and Ireland ( Asher *et al.*, 2013; Harper *et al.*, 2002; GBIF Secretariat, 2022; Sterling & Parsons, 2018).

The adult moth has grey-brown wings with black spots and an overall shiny appearance, and is commonly found resting on walls inside houses at dusk. The larvae are omnivorous, capable of feeding on woollen fabrics, book bindings and leather or on a variety of food stuffs including stored grains; in domestic situations the species more commonly feeds on detritus and dead skin found behind skirting boards or in dusty spaces ( Harper *et al.*, 2002). It can also be abundant in old birds’ nests ( Boyes & Lewis, 2019). Although the species has been described as bivoltine, the life-cycle is highly dependent on environmental conditions: in laboratory culture the complete life-cycle varies from 6 to 14 months ( Woodroffe, 1951). This variability is partly due to ability of the egg to arrest for up to 100 days if conditions are not optimal and also due to a flexible length larval diapause phase ( Woodroffe, 1951). In centrally-heated houses in the UK, the adult moth is seen primarily from May to September ( Asher *et al.*, 2013; NBN Atlas Partnership, 2022).

The biochemical basis of the ability to digest keratin by *H. pseudospretella* is not fully understood. It is possible that the property is conferred by cobiont microorganisms and consistent with this suggestion several bacterial species have been isolated from the larval midgut, although the functional biology of these bacteria has not been elucidated ( Shannon *et al.*, 2001). Furthermore, the bacteria identified to date do not seem specifically adapted for the reducing conditions present in the larval gut ( Shannon *et al.*, 2001), nor were crypts or other specialised bacterial-holding ‘organs’ found ( Gerard, 2002).

Here we present a complete genome sequence for the Brown House-moth *H. pseudospretella*. The assembled genome sequence will facilitate attempts to understand the highly flexible life-history of this species and its adaptations to an omnivorous diet.

## Genome sequence report

**Figure 1.**
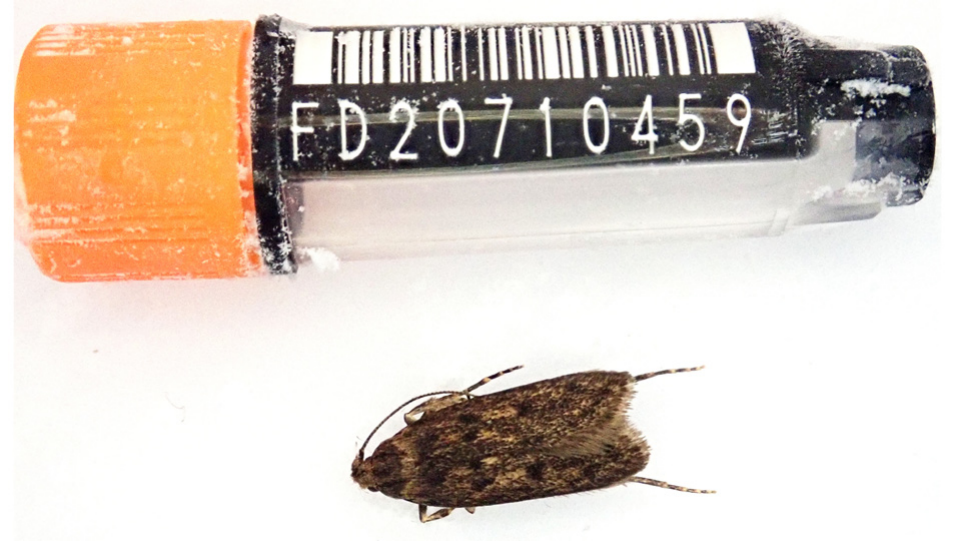
Photograph of the *Hofmannophila pseudospretella* (ilHofPseu3) specimen used for genome sequencing.

The genome was sequenced from one male *Hofmannophila pseudospretella* ( Figure 1) collected from Wytham Woods, Oxfordshire (biological vice-county: Berkshire), UK (latitude 51.77, longitude –1.34). A total of 58-fold coverage in Pacific Biosciences single-molecule HiFi long reads was generated. Primary assembly contigs were scaffolded with chromosome conformation Hi-C data. Manual assembly curation corrected 12 missing joins or mis-joins, reducing the scaffold count by three.

**Figure 2.**
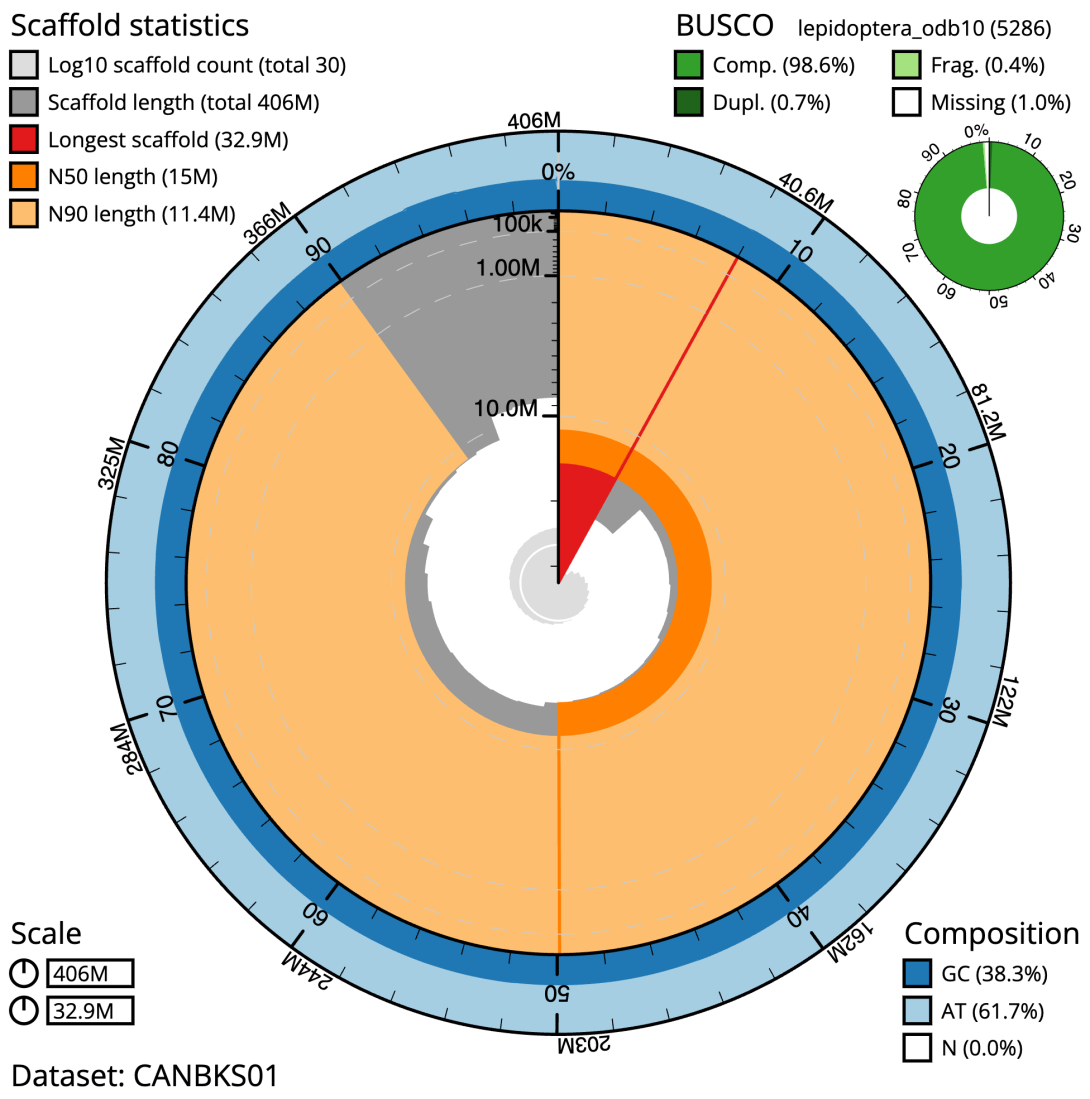
Genome assembly of *Hofmannophila pseudospretella*, ilHofPseu3.1: metrics. The BlobToolKit Snailplot shows N50 metrics and BUSCO gene completeness. The main plot is divided into 1,000 size-ordered bins around the circumference with each bin representing 0.1% of the 406,170,397 bp assembly. The distribution of scaffold lengths is shown in dark grey with the plot radius scaled to the longest scaffold present in the assembly (32,859,644 bp, shown in red). Orange and pale-orange arcs show the N50 and N90 scaffold lengths (14,950,471 and 11,363,009 bp), respectively. The pale grey spiral shows the cumulative scaffold count on a log scale with white scale lines showing successive orders of magnitude. The blue and pale-blue area around the outside of the plot shows the distribution of GC, AT and N percentages in the same bins as the inner plot. A summary of complete, fragmented, duplicated and missing BUSCO genes in the lepidoptera_odb10 set is shown in the top right. An interactive version of this figure is available at https://blobtoolkit.genomehubs.org/view/ilHofPseu3.1/dataset/CANBKS01/snail.

The final assembly has a total length of 406.2 Mb in 30 sequence scaffolds with a scaffold N50 of 15.0 Mb ( Table 1). Most (99.97%) of the assembly sequence was assigned to 28 chromosomal-level scaffolds, representing 27 autosomes, and the Z sex chromosome. Chromosome-scale scaffolds confirmed by the Hi-C data are named in order of size ( Figure 2– Figure 5; Table 2). While not fully phased, the assembly deposited is of one haplotype. Contigs corresponding to the second haplotype have also been deposited. The mitochondrial genome was also assembled and can be found as a contig within the multifasta file of the genome submission.

**Table 1.** Genome data for *Hofmannophila pseudospretella*, ilHofPseu3.1.

Project accession data		
Assembly identifier	ilHofPseu3.1	
Species	*Hofmannophila pseudospretella*	
Specimen	ilHofPseu3	
NCBI taxonomy ID	572861	
BioProject	PRJEB56489	
BioSample ID	SAMEA10979205	
Isolate information	ilHofPseu3	
Assembly metrics *		*Benchmark*
Consensus quality (QV)	66	*≥ 50*
*k*-mer completeness	100%	*≥ 95%*
BUSCO **	C:98.6%[S:98.0%,D:0.7%], F:0.4%,M:1.0%,n:5,286	*C ≥ 95%*
Percentage of assembly mapped to chromosomes	99.97%	*≥ 95%*
Sex chromosomes	Z chromosome	*localised homologous pairs*
Organelles	Mitochondrial genome assembled.	*complete single alleles*
Raw data accessions		
PacificBiosciences SEQUEL II	ERR10357396	
Hi-C Illumina	ERR10323143	
Genome assembly		
Assembly accession	GCA_947369225.1	
*Accession of alternate haplotype*	GCA_947369255.1	
Span (Mb)	406.2	
Number of contigs	80	
Contig N50 length (Mb)	8.6	
Number of scaffolds	30	
Scaffold N50 length (Mb)	15.0	
Longest scaffold (Mb)	32.9	

* Assembly metric benchmarks are adapted from column VGP-2020 of “Table 1: Proposed standards and metrics for defining genome assembly quality” from ( Rhie *et al.*, 2021). ** BUSCO scores based on the lepidoptera_odb10 BUSCO set using v5.3.2. C = complete [S = single copy, D = duplicated], F = fragmented, M = missing, n = number of orthologues in comparison. A full set of BUSCO scores is available at https://blobtoolkit.genomehubs.org/view/ilHofPseu3.1/dataset/CANBKS01/busco.

**Figure 3.**
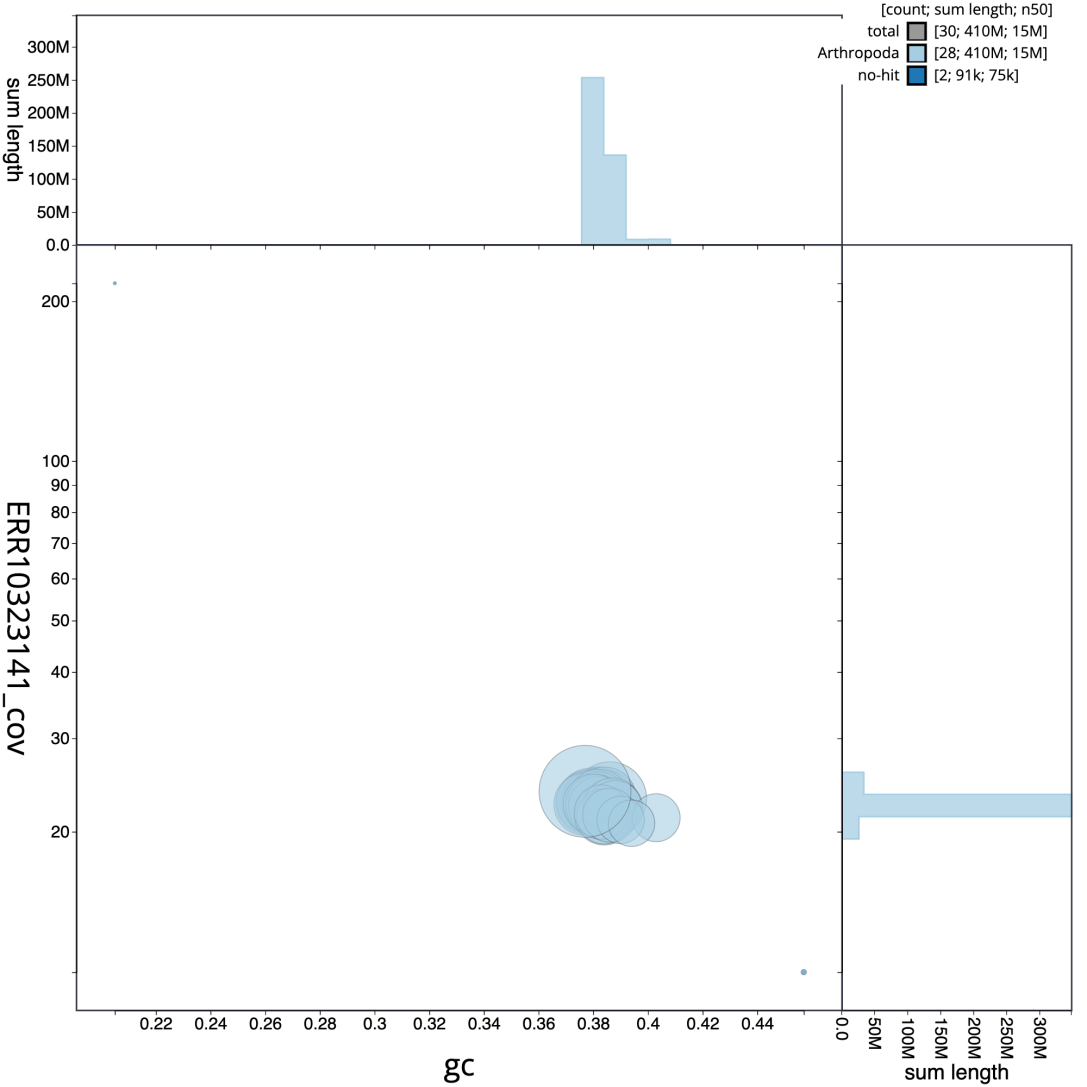
Genome assembly of *Hofmannophila pseudospretella*, ilHofPseu3.1: GC coverage. BlobToolKit GC-coverage plot. Scaffolds are coloured by phylum. Circles are sized in proportion to scaffold length. Histograms show the distribution of scaffold length sum along each axis. An interactive version of this figure is available at https://blobtoolkit.genomehubs.org/view/ilHofPseu3.1/dataset/CANBKS01/blob.

**Figure 4.**
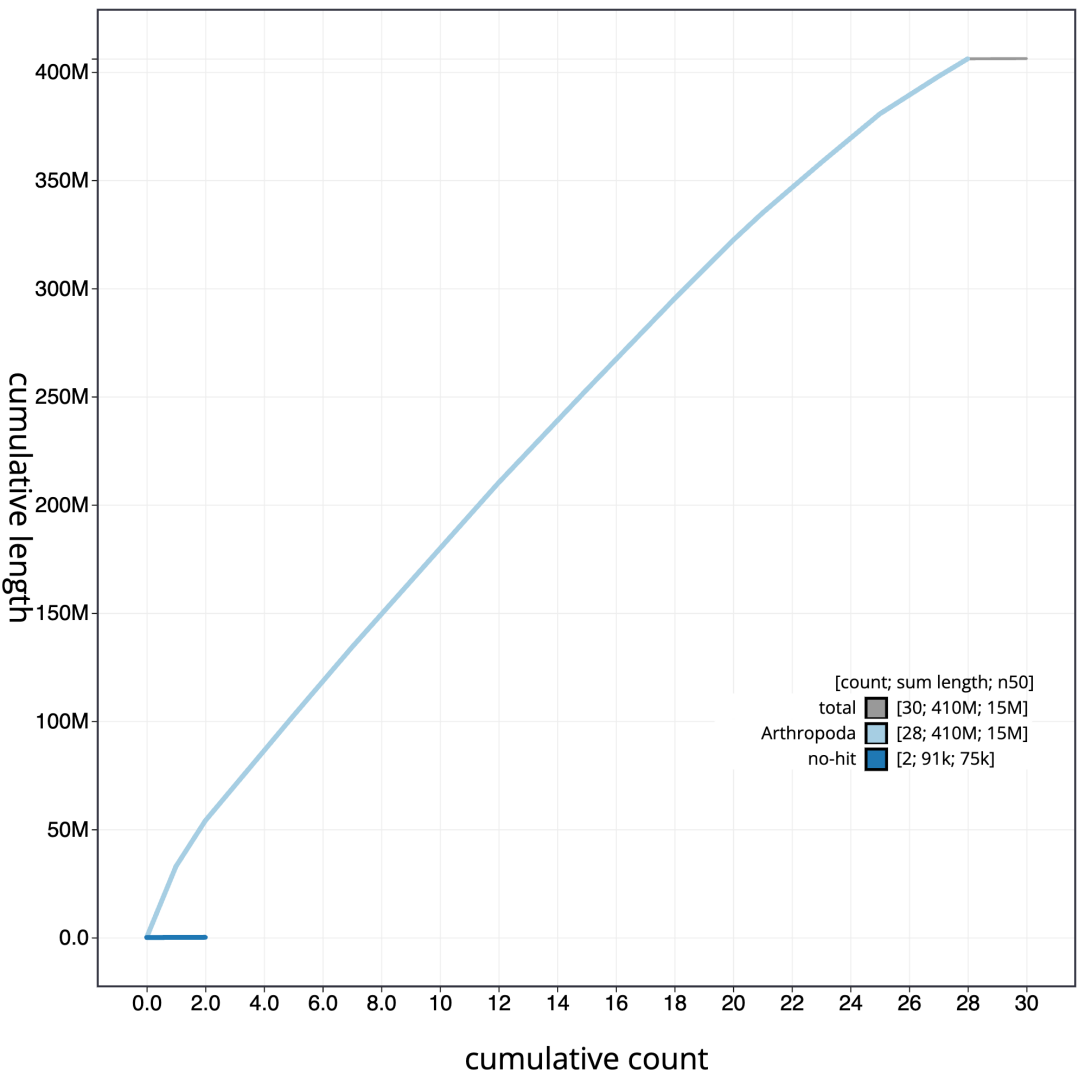
Genome assembly of *Hofmannophila pseudospretella*, ilHofPseu3.1: cumulative sequence. BlobToolKit cumulative sequence plot. The grey line shows cumulative length for all scaffolds. Coloured lines show cumulative lengths of scaffolds assigned to each phylum using the buscogenes taxrule. An interactive version of this figure is available at https://blobtoolkit.genomehubs.org/view/ilHofPseu3.1/dataset/CANBKS01/cumulative.

**Figure 5.**
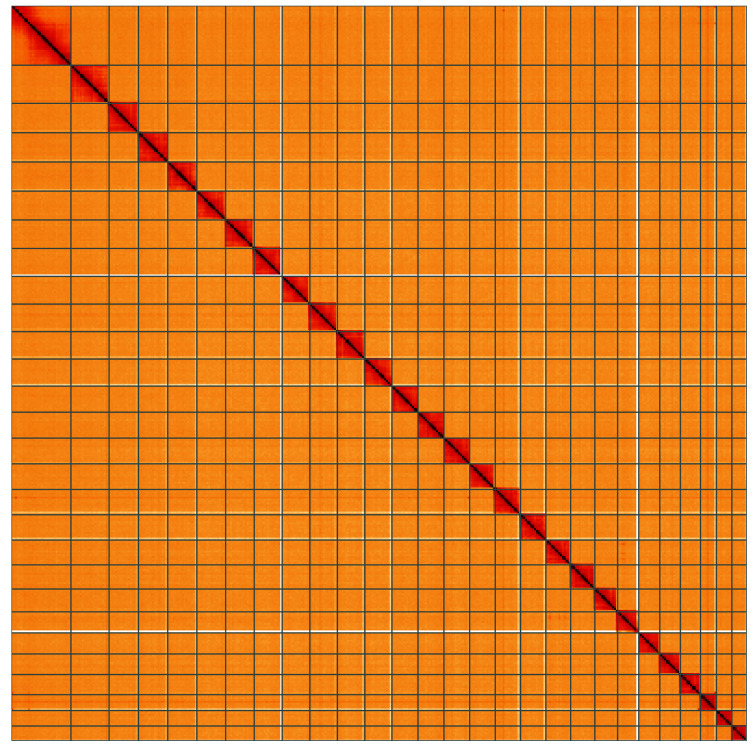
Genome assembly of *Hofmannophila pseudospretella*, ilHofPseu3.1: Hi-C contact map. Hi-C contact map of the ilHofPseu3.1 assembly, visualised using HiGlass. Chromosomes are shown in order of size from left to right and top to bottom. An interactive version of this figure may be viewed at https://genome-note-higlass.tol.sanger.ac.uk/l/?d=ee6b4s9ITASMhI7KhzAbAA.

**Table 2.** Chromosomal pseudomolecules in the genome assembly of *Hofmannophila pseudospretella*, ilHofPseu3.

INSDC accession	Chromosome	Size (Mb)	GC%
OX376312.1	1	21.09	38.6
OX376313.1	2	16.23	38.2
OX376314.1	3	16.15	38.4
OX376315.1	4	16.09	38.4
OX376316.1	5	15.93	37.9
OX376317.1	6	15.77	37.8
OX376318.1	7	15.4	38
OX376319.1	8	15.31	38
OX376320.1	9	15.24	38.2
OX376321.1	10	15.12	37.8
OX376322.1	11	14.95	37.7
OX376323.1	12	14.41	38.1
OX376324.1	13	14.31	38.2
OX376325.1	14	14.21	38.2
OX376326.1	15	14.15	38.1
OX376327.1	16	13.99	38.2
OX376328.1	17	13.97	38
OX376329.1	18	13.7	38.4
OX376330.1	19	13.34	38.7
OX376331.1	20	12.61	38.7
OX376332.1	21	11.65	38.8
OX376333.1	22	11.6	38.4
OX376334.1	23	11.36	38.3
OX376335.1	24	11.16	38.6
OX376336.1	25	8.74	40.3
OX376337.1	26	8.56	39
OX376338.1	27	8.19	39.4
OX376311.1	Z	32.86	37.7
OX376339.1	MT	0.02	20.7

The estimated Quality Value (QV) of the final assembly is 66 with *k*-mer completeness of 100%, and the assembly has a BUSCO v5.3.2 completeness of 98.6% (single = 98.0%, duplicated = 0.7%), using the lepidoptera_odb10 reference set ( *n* = 5,286).

Metadata for specimens, spectral estimates, sequencing runs, contaminants and pre-curation assembly statistics can be found at https://links.tol.sanger.ac.uk/species/572861.

## Methods

### Sample acquisition and nucleic acid extraction

A male *Hofmannophila pseudospretella* (individual ilHofPse3, specimen Ox001942) was collected from Wytham Woods, Oxfordshire (biological vice-county: Berkshire), UK (latitude 51.77, longitude –1.34) by Douglas Boyes (University of Oxford). The specimen was reared from birds’ nest material collected after juvenile birds had fledged in late spring 2021; the adult moth was preserved on dry ice on 16 June 2021.

The ilHofPseu3 sample was prepared by the Tree of Life laboratory, Wellcome Sanger Institute (WSI). The sample was weighed and dissected on dry ice with tissue set aside for Hi-C sequencing. Whole organism tissue was disrupted using a Nippi Powermasher fitted with a BioMasher pestle. DNA was extracted at the Wellcome Sanger Institute (WSI) Scientific Operations core using the Qiagen MagAttract HMW DNA kit, according to the manufacturer’s instructions.

### Sequencing

Pacific Biosciences HiFi circular consensus DNA sequencing libraries were constructed according to the manufacturers’ instructions. DNA sequencing was performed by the Scientific Operations core at the WSI on Pacific Biosciences SEQUEL II (HiFi) instrument. Hi-C data were also generated from tissue of ilHofPse3 that had been set aside, using the Arima2 kit and sequenced on the Illumina NovaSeq 6000 instrument.

### Genome assembly, curation and evaluation

Assembly was carried out with Hifiasm ( Cheng *et al.*, 2021) and haplotypic duplication was identified and removed with purge_dups ( Guan *et al.*, 2020). The assembly was then scaffolded with Hi-C data ( Rao *et al.*, 2014) using YaHS ( Zhou *et al.*, 2023). The assembly was checked for contamination and corrected using the gEVAL system ( Chow *et al.*, 2016) as described previously ( Howe *et al.*, 2021). Manual curation was performed using gEVAL, HiGlass ( Kerpedjiev *et al.*, 2018) and Pretext ( Harry, 2022). The mitochondrial genome was assembled using MitoHiFi ( Uliano-Silva *et al.*, 2022), which runs MitoFinder ( Allio *et al.*, 2020) or MITOS ( Bernt *et al.*, 2013) and uses these annotations to select the final mitochondrial contig and to ensure the general quality of the sequence. To evaluate the assembly, MerquryFK was used to estimate consensus quality (QV) scores and *k*-mer completeness ( Rhie *et al.*, 2020). The genome was analysed within the BlobToolKit environment ( Challis *et al.*, 2020) and BUSCO scores ( Manni *et al.*, 2021; Simão *et al.*, 2015) were calculated. Table 3 contains a list of software tool versions and sources.

**Table 3.** Software tools: versions and sources.

Software tool	Version	Source
BlobToolKit	4.0.7	https://github.com/blobtoolkit/blobtoolkit
BUSCO	5.3.2	https://gitlab.com/ezlab/busco
gEVAL	N/A	https://geval.org.uk/
Hifiasm	0.16.1-r375	https://github.com/chhylp123/hifiasm
HiGlass	1.11.6	https://github.com/higlass/higlass
Merqury	MerquryFK	https://github.com/thegenemyers/MERQURY.FK
MitoHiFi	2	https://github.com/marcelauliano/MitoHiFi
PretextView	0.2	https://github.com/wtsi-hpag/PretextView
purge_dups	1.2.3	https://github.com/dfguan/purge_dups
YaHS	yahs-1.1.91eebc2	https://github.com/c-zhou/yahs

### Ethics and compliance issues

The materials that have contributed to this genome note have been supplied by a Darwin Tree of Life Partner. The submission of materials by a Darwin Tree of Life Partner is subject to the Darwin Tree of Life Project Sampling Code of Practice. By agreeing with and signing up to the Sampling Code of Practice, the Darwin Tree of Life Partner agrees they will meet the legal and ethical requirements and standards set out within this document in respect of all samples acquired for, and supplied to, the Darwin Tree of Life Project. All efforts are undertaken to minimise the suffering of animals used for sequencing. Each transfer of samples is further undertaken according to a Research Collaboration Agreement or Material Transfer Agreement entered into by the Darwin Tree of Life Partner, Genome Research Limited (operating as the Wellcome Sanger Institute), and in some circumstances other Darwin Tree of Life collaborators.

## Data Availability

European Nucleotide Archive: *Hofmannophila pseudospretella* (brown house moth). Accession number PRJEB56489; https://identifiers.org/ena.embl/PRJEB56489. ( Wellcome Sanger Institute, 2022) The genome sequence is released openly for reuse. The *Hofmannophila pseudospretella* genome sequencing initiative is part of the Darwin Tree of Life (DToL) project. All raw sequence data and the assembly have been deposited in INSDC databases. The genome will be annotated using available RNA-Seq data and presented through the Ensembl pipeline at the European Bioinformatics Institute. Raw data and assembly accession identifiers are reported in Table 1.

## Author information

Members of the University of Oxford and Wytham Woods Genome Acquisition Lab are listed here: https://doi.org/10.5281/zenodo.4789928.

Members of the Darwin Tree of Life Barcoding collective are listed here: https://doi.org/10.5281/zenodo.4893703.

Members of the Wellcome Sanger Institute Tree of Life programme are listed here: https://doi.org/10.5281/zenodo.4783585.

Members of Wellcome Sanger Institute Scientific Operations: DNA Pipelines collective are listed here: https://doi.org/10.5281/zenodo.4790455.

Members of the Tree of Life Core Informatics collective are listed here: https://doi.org/10.5281/zenodo.5013541.

Members of the Darwin Tree of Life Consortium are listed here: https://doi.org/10.5281/zenodo.4783558.
