# Supplementary material for: The genome sequence of the Brown House-moth, Hofmannophila pseudospretella (Stainton, 1849)
Source: Wellcome Open Res. 2023 May 31;8:230. [Version 1] doi: 10.12688/wellcomeopenres.19394.1 (PMC12457894; doi:10.12688/wellcomeopenres.19394.1)
Supplement: Peer review history

# Reviewer report: Reviewer response for version 1

**DOI:** 10.21956/wellcomeopenres.21482.r128869

**Published:** 2025-09-23

**Authors:** Marko Mutanen

**Affiliations:** 1 University of Oulu, Oulu, Finland

This is a well-prepared genome report with a nice introduction to the species of concern, Hofmannophila pseudospretella. Indeed, the article gives a rather broad overview for the unusual life history of this species. This introduction however does not touch the systematics or classification of this species which is a rather isolated among the members of its family. There is published data about its phylogenetic position.

 The genome that comes from a male specimen is clearly of high quality, as indicated by numerous metrics and graphs provided. For example, the USCO coverage is high, and N50 and N90 values are high too. The genome has high potential to be used in future works of e.g. phylogenomics and diet evolution studies.

Are sufficient details of methods and materials provided to allow replication by others?

Yes

Is the rationale for creating the dataset(s) clearly described?

Yes

Are the datasets clearly presented in a useable and accessible format?

Yes

Are the protocols appropriate and is the work technically sound?

Yes

Reviewer Expertise:

Phylogenetics, DNA barcoding, molecular taxonomy, dark taxa, species delimitation

I confirm that I have read this submission and believe that I have an appropriate level of expertise to confirm that it is of an acceptable scientific standard.

# Reviewer report: Reviewer response for version 1

**DOI:** 10.21956/wellcomeopenres.21482.r128866

**Published:** 2025-09-02

**Authors:** Sivasankaran Kuppusamy

**Affiliations:** 1 Loyola College, Chennai, Tamil Nadu, India

Authors have done the chromosome-level genome assembly of *Hofmannophila pseudospretella *(Stainton, 1849). They used two platforms (Illumina NovaSeq and SEQUEL II (HiFi)) for the sequencing. Totally 406.2 megabases genome sequence was received from the genome assembly. Authors have used appropriate software for the sequence assembly and genome annotations. Furthermore, 28 chromosomal pseudomolecules including sex chromosome were scaffolded from the assembly.

 Minor comments on the manuscript

 The length of the genome sequencing has not been given by the authors. the whole length genome sequence can be provided in the table.

 The assembly yielded a genome of 406.2 megabases in total. Gene transcripts, non-coding genes, and protein-coding genes can be detected using genome annotation. The authors haven't provided the annotation details. Any reason?

 The research article was well prepared and the manuscript meets the necessary scientific standard and is suitable for indexing

Are sufficient details of methods and materials provided to allow replication by others?

Yes

Is the rationale for creating the dataset(s) clearly described?

Yes

Are the datasets clearly presented in a useable and accessible format?

Yes

Are the protocols appropriate and is the work technically sound?

Yes

Reviewer Expertise:

Phylogenetic analysis of Noctuoidea moths using mitochondrial genome

I confirm that I have read this submission and believe that I have an appropriate level of expertise to confirm that it is of an acceptable scientific standard.

# Reviewer report: Reviewer response for version 1

**DOI:** 10.21956/wellcomeopenres.21482.r107252

**Published:** 2024-11-20

**Authors:** Fahad Alqahtani

**Affiliations:** 1 King Abdulaziz City for Science and Technology, Riyadh, Saudi Arabia

The authors of "The genome sequence of the Brown House-moth, *Hofmannophila pseudospretella* (Stainton, 1849)" have successfully produced a highly complete genome assembly at the chromosome level for a male Brown House-moth ( *Hofmannophila pseudospretella*). The genome assembly was carried out using two sequencing technologies: Pacific Biosciences Sequel II and Hi-C Illumina. The final assembly has a total length of 406.2 Mb and contains 28 chromosomes. The completeness of the genome assembly was evaluated using BUSCO analysis, which showed that 98.6% of the common genes were completely present in the lepidoptera_odb10 dataset.

 However, there are a few minor issues that should be addressed:

- The number of contigs is reported as 80, which slightly differs from the number listed in NCBI (79). This discrepancy should be clarified to ensure consistency.
- The number of scaffolds is reported as 30, while NCBI lists it as 29. This difference should be addressed for consistency.
- The GC content for most of the chromosomes differs slightly from the value listed in NCBI. This discrepancy should be clarified to ensure consistency.
- In the "Genome assembly, curation, and evaluation" section, the authors misspell the name of the tool "Pretext." It should be "PretextView," as shown in Table 3.

Are sufficient details of methods and materials provided to allow replication by others?

Yes

Is the rationale for creating the dataset(s) clearly described?

Yes

Are the datasets clearly presented in a useable and accessible format?

Yes

Are the protocols appropriate and is the work technically sound?

Yes

Reviewer Expertise:

Bioinformatics

I confirm that I have read this submission and believe that I have an appropriate level of expertise to confirm that it is of an acceptable scientific standard, however I have significant reservations, as outlined above.

# Response

**Published:** 2024-11-20

**Authors:** Tree of Life Team Sanger

**Affiliations:** Wellcome Trust Sanger Institute, UK

Thank you for your review. 

 The difference of 1 in the reported numbers of scaffolds and contigs is that the mitochondrial genome is not considered when reporting the nuclear assembly.
